# The effect of two ribonucleases on the production of Shiga toxin and *stx*-bearing bacteriophages in Enterohaemorrhagic *Escherichia coli*

**DOI:** 10.1038/s41598-021-97736-z

**Published:** 2021-09-15

**Authors:** Patricia B. Lodato

**Affiliations:** Department of Microbiology and Immunology, Kirksville College of Osteopathic Medicine, A.T. Still University, Kirksville, MO 63501 USA

**Keywords:** Microbiology, Bacterial pathogenesis

## Abstract

Enterohaemorrhagic *Escherichia coli* (EHEC) comprise a group of intestinal pathogens responsible for a range of illnesses, including kidney failure and neurological compromise. EHEC produce critical virulence factors, Shiga toxin (Stx) 1 or 2, and the synthesis of Stx2 is associated with worse disease manifestations. Infected patients only receive supportive treatment because some conventional antibiotics enable toxin production. Shiga toxin 2 genes (*stx2*) are carried in λ-like bacteriophages (*stx2*-phages) inserted into the EHEC genome as prophages. Factors that cause DNA damage induce the lytic cycle of *stx2*-phages, leading to Stx2 production. The phage Q protein is critical for transcription antitermination of *stx2* and phage lytic genes. This study reports that deficiency of two endoribonucleases (RNases), E and G, significantly delayed cell lysis and impaired production of both Stx2 and *stx2*-phages, unlike deficiency of either enzyme alone. Moreover, scarcity of both enzymes reduced the concentrations of *Q* and *stx2* transcripts and slowed cell growth.

## Introduction

Enterohaemorrhagic *Escherichia coli* (EHEC) comprise a group of enteric pathogens that cause a spectrum of clinical manifestations, including diarrhea, bloody diarrhea or the more serious hemolytic uremic syndrome (HUS), and multi-organ disease^1^. A critical virulence trait of EHEC is the production of Shiga toxin^2,3^. Stx primarily targets renal microvascular endothelial cells, but other cell types such as neurons are also susceptible^4,5^. There is no available antidote to neutralize the toxin, which inhibits protein synthesis by cleaving the N-glycosidic bond at adenine 4324 in 28S ribosomal RNA^6^.

There are two immunologically distinct Stx types, Stx1 and Stx2. The Stx1 and Stx2 types are further classified into three and seven well-known subtypes, respectively^7^. The presence of Stx2-encoding genes (*stx2* genes) and the production of Stx2 have been linked to worse disease outcomes in animal models and epidemiological studies^8–11^. In addition, the Stx2a subtype is highly associated with the development of HUS^12^. The *stx2* genes are located in the genome of resident lambdoid prophages, which share a similar developmental cycle with the λ phage of nonpathogenic *E. coli*^13,14^. The *stx2*-harboring phages (*stx2*-phages) usually exist as prophages, but exposure to DNA-damaging agents, such as mitomycin C (MMC), induces the initiation of the lytic growth cycle^15–17^. After DNA damage occurs, the cell DNA damage response is triggered, resulting in an increase in the transcription of the host *recA* gene^18,19^ and the formation of single-stranded DNA-RecA complexes. These complexes mediate the self-cleavage of the phage transcriptional repressor cI^20^, resulting in transcription of additional phage genes, among them, the gene *Q*^14^. The Q protein acts as a transcriptional antiterminator that allows read-through of a terminator leading to *stx2* expression^21^. In the final step of the lytic cycle, the bacterial cells lyse and release phage particles and toxin outside the cell^22^. While Stx reaches systemic circulation and the target organs, the *stx* phages can potentially infect phage-sensitive bacteria, contributing to the dissemination of *stx* genes to new hosts^23–26^.

The prevention of EHEC transmission is currently the best strategy to avoid infection and disease^27^. The administration of conventional antibiotics is not recommended, and available treatment options rely on fluids, electrolyte management, and dialysis in cases of kidney failure^28–30^. Some antibiotics (e.g., quinolones, trimethoprim-sulfamethoxazole) induce the *stx*-phage lytic cycle and high Stx production^31–33^. However, in vitro and in vivo studies indicate that other antibiotics (e.g., rifaximin, azithromycin) do not trigger the synthesis of toxin in certain Stx-producing *E. coli*^33–36^.

Enzymes involved in RNA turnover, such as the well-characterized endoribonuclease (RNase) E, influence the expression of virulence traits^37,38^ and have been proposed as potential antibacterial targets^39^. RNase E is present in Gram-negative bacteria and has a central role in degradation of mRNAs, cleavage of precursor rRNAs and tRNAs, and degradation or cleavage of small regulatory RNAs^40^. The RNase E N-terminal catalytic domain is essential and shares about 32% sequence identity with its homolog RNase G. In contrast to RNase E, RNase G is dispensable for viability^41^. Despite some differences in substrate requirements, both enzymes prefer 5’ end-monophosphorylated RNA and attack single-stranded AU-rich sequences^42–44^. In *E. coli*, RNase G cleaves the 5’ end of a 16S rRNA precursor^42^ and controls the stability of a limited number of mRNAs^45–47^. However, RNase G may have a global role in mRNA stability in some bacteria other than *E. coli*^48^. Because RNase E is required for viability, advances concerning its role in virulence have been hampered. To overcome this impediment, we previously reported the construction of an EHEC strain in which RNase E synthesis is controlled by addition of a chemical (isopropyl β-D-1-thiogalactopyranoside [IPTG]) to the culture medium^49^. Depletion of RNase E produced lower *stx2*-phage yields and an initial delay in Stx2 production; however, final Stx2 concentrations were as high as the control^50^. In this study, the RNase G-encoding gene (*rng*) was deleted and its effect on the production of *stx2*-phages and Stx2 was examined under normal and deficient RNase E backgrounds.

## Results

### Growth profile of EHEC with deficiency in RNase E, RNase G, or both enzymes in MMC-treated cultures

The RNase G-encoding gene (*rng*) was deleted in the EHEC strains TEA028 (parental) and TEA028-*rne* to generate the strains TEA028-Δ*rng* and TEA028-*rne*-Δ*rng*, respectively (Supplementary Table S1). RNase E is IPTG-inducible in TEA028-*rne*, producing normal levels of RNase E in medium supplemented with 100 µM IPTG, but low levels of RNase E at or below 1 µM IPTG^49,50^. Deletion of the *rng* gene in TEA028-*rne*-Δ*rng* did not change the inducible expression pattern of RNase E (Supplementary Fig. S1).

To induce the *stx2*-phage lytic cycle, cultures of the TEA028 parental strain and its RNase E and G derivatives were treated with subinhibitory concentrations of MMC (1 µg/mL). The strains were grown to optical density at 600 nm (OD_600_) of 0.30–0.35 (time 0), and then MMC was added to an aliquot of the cultures. Thereafter, the turbidity of the cultures was determined at various time intervals (Supplementary Fig. S2).

**Figure 1:**
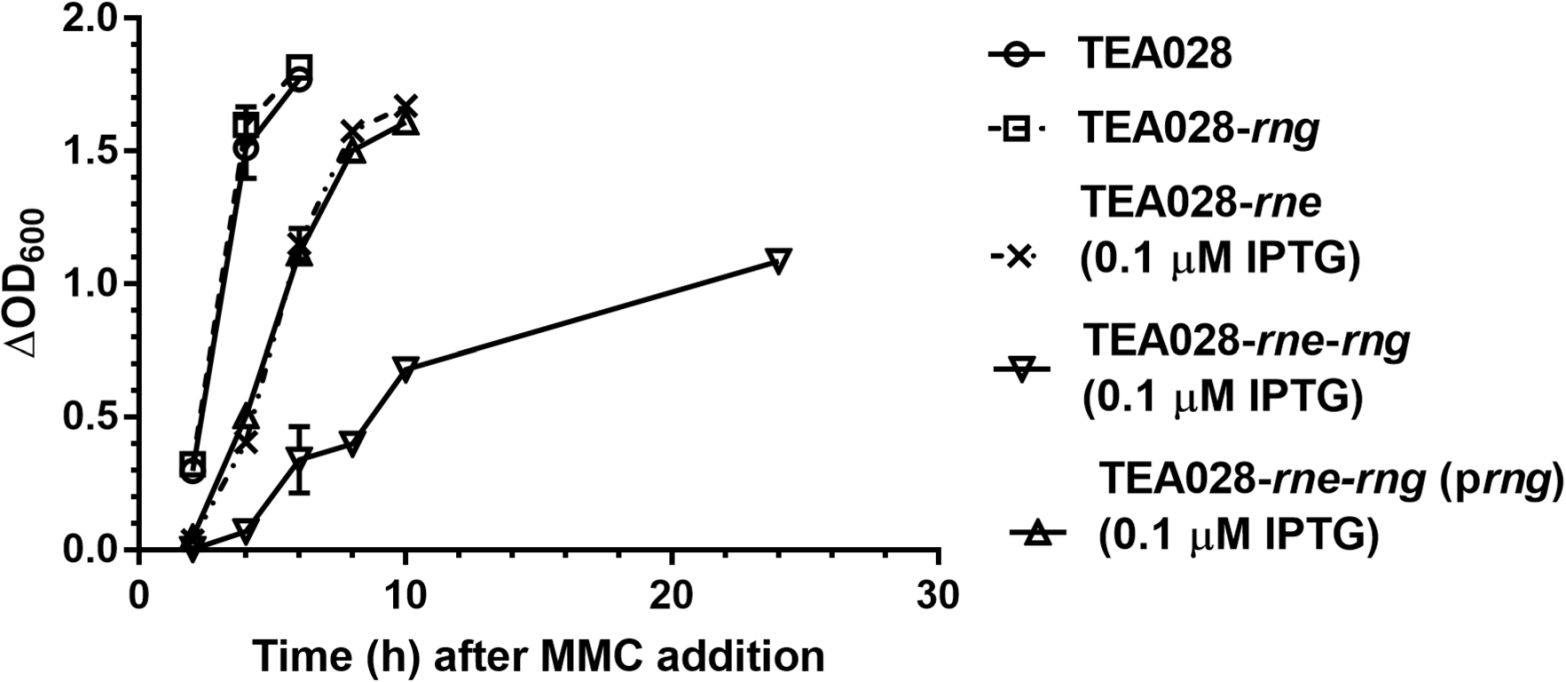
Growth of EHEC TEA028 and its RNases E and G derivative strains in cultures treated or non-treated with mitomycin C (MMC). Cultures of TEA028 (parental strain) and its RNases E and G derivatives were grown in Luria Bertani medium to optical density at 600 nm (OD_600_) of 0.30–0.35 at which point the cultures were split and an aliquot was treated with MMC (1 µg/mL) to induce the *stx2*-phage lytic cycle. Thereafter, samples were collected at various time points to measure OD_600_. The strains TEA028-*rne* and TEA028-*rne*-Δ*rng* underproduce RNase E when the medium is supplemented with low levels of isopropyl β-d-1-thiogalactopyranoside (IPTG), as indicated. For each time point, the OD_600_ of MMC-treated cultures was subtracted from the OD_600_ of non-treated aliquots (ΔOD_600_). Means and standard errors or at least 4 biological replicates are graphed. Lack of error bars indicates that the standard error was smaller than the plot symbol.

The difference of turbidity readings between MMC-treated and non-treated cultures is a measure of cell lysis progression after MMC addition due to activation of the phage lytic cycle. As reported previously^50^, RNase E deficiency resulted in slower rate of lysis (Fig. 1, compare TEA028 cells vs. TEA028-*rne* cells at 0.1 µM IPTG). The absence of RNase G in RNase E-deficient cells (TEA028-*rne*-Δ*rng* at 0.1 µM IPTG) provoked a substantial delay in cell lysis; in contrast, the absence of RNase G alone (TEA028-Δ*rng*) had no effect. Complementation of the *rng* deletion with the *rng* gene expressed from a plasmid in the TEA028-*rne*-Δ*rng* (p*rng*) strain resulted in a similar rate of cell lysis to TEA028-*rne.*

Supplementation with 100 µM IPTG did not affect significantly the response to MMC treatment of the strains TEA028 or TEA028-Δ*rng* (Supplementary Fig. S3). Likewise, the strains TEA028-*rne* at 100 µM IPTG (normal levels of RNase E) and the parental strain TEA028 behaved similarly after MMC addition (Supplementary Fig. S4) in agreement with our previous results^50^.

The delayed kinetics of cell lysis after induction of the phage lytic cycle suggested that deficiency of RNases E and G could impair the production of *stx2-*phages, Stx2, or both.

### Production of *stx*-phages in EHEC with deficiency in RNase E, RNase G, or both enzymes in MMC-treated cultures

**Figure 2:**
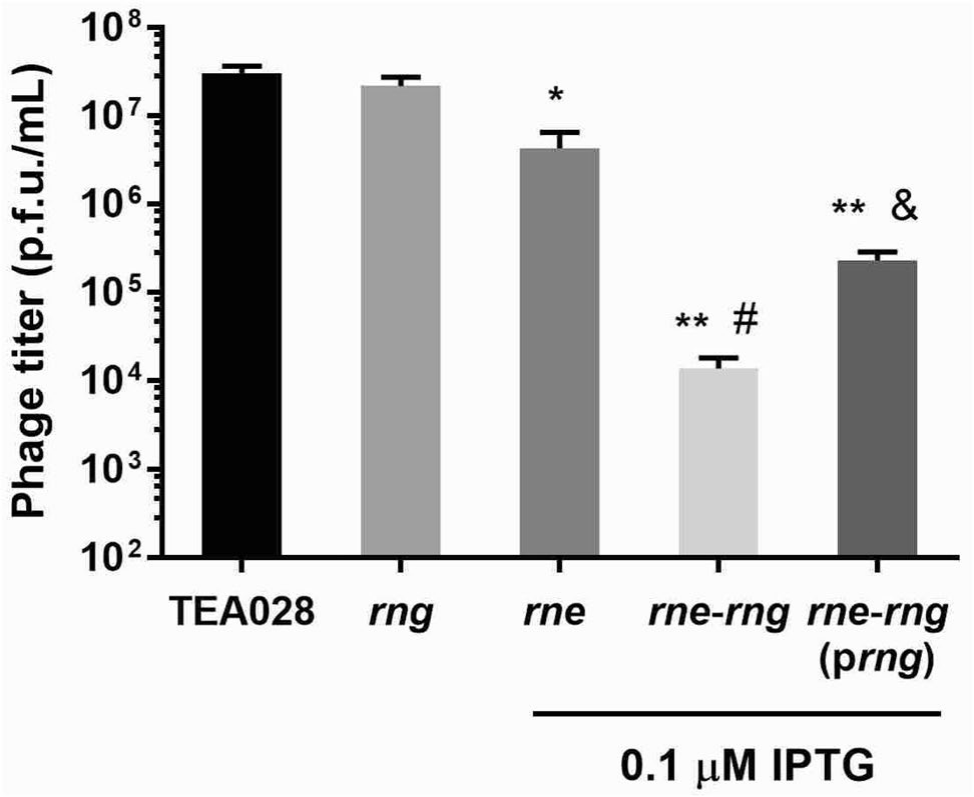
Production of phages by EHEC containing normal levels of RNases E and G versus derivative strains deficient in either or both enzymes. Cultures of TEA028 (parental strain) and its RNases E and G derivatives were grown in LB medium to an optical density at 600 nm of 0.30–0.35 at which point the cultures were treated with MMC (1 µg/mL) to induce the phage lytic cycle. Supernatants were collected at 6 h, and phage titers were determined by the double agar assay as described in “Methods” section. The strains TEA028-*rne*, TEA028-*rne*-Δ*rng*, and TEA028-*rne*-Δ*rng* (p*rng*) underproduce RNase E when the medium is supplemented with 0.1 µM IPTG. Means and standard errors of 3–4 biological replicates are shown. Abbreviations: *rng*: TEA028-Δ*rng*; *rne*: TEA028-*rne*; *rne-rng*: TEA028-*rne*-Δ*rng*; *rne*-*rng* (p*rng*): TEA028-*rne*-Δ*rng* (p*rng*); p.f.u.: plaque forming units. * Adjusted *P* value versus TEA028 = 0.0095; ** adjusted *P* value versus TEA028 = 0.0001; # adjusted *P* value versus TEA028-*rne* = 0.0007; & adjusted *P* value versus TEA028-*rne-*Δ*rng* = 0.0015.

Phage yields were determined in culture supernatants of TEA028 and its RNases E and G derivatives after MMC-treatment for 6 h. After treatment with MMC, EHEC TEA028 produces *stx2*-phages, which were detected by plaque hybridization under the same experimental conditions in a previous study^50^; however, plaques of *stx1*-phages were not detected. RNase E deficiency (TEA028-*rne* at 0.1 µM IPTG) impaired the production of infectious phages in agreement with our previous findings (Fig. 2) while TEA028-*rne* at 100 µM IPTG (normal RNase E levels) produced similar phage yields to the control (Supplementary Fig. S4). Deficiency of both RNase E and RNase G (TEA028-*rne*-Δ*rng* at 0.1 µM IPTG) reduced phage yields even further, whereas the absence of only RNase G caused no effect. Complementation of the *rng* deletion partially restored the production of phages (TEA028-*rne*-Δ*rng* (p*rng*) vs. TEA028-*rne*-Δ*rng* at 0.1 µM IPTG).

### Production of toxin in EHEC with deficiency in RNase E, RNase G, or both enzymes in MMC-treated cultures

**Figure 3:**
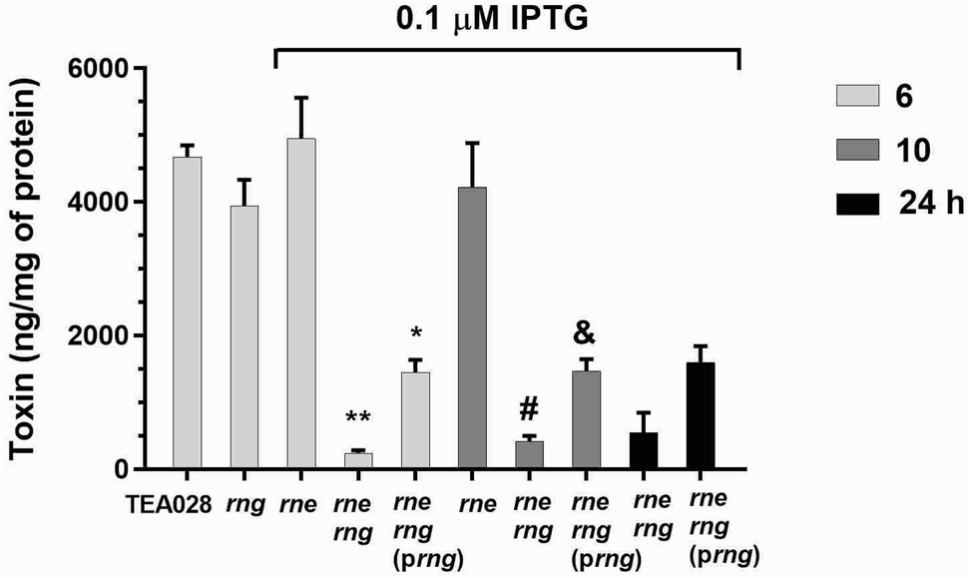
Toxin (Stx2a subtype) production by EHEC containing normal levels of RNases E and G versus derivative strains deficient in either or both enzymes. Cultures of TEA028 (parental strain) and its RNase E/G derivatives were grown in LB medium to an optical density at 600 nm of 0.30–0.35 at which point the cultures were treated with MMC (1 µg/mL) to induce the phage lytic cycle and toxin production. Thereafter, samples were collected at the indicated time intervals, and toxin concentrations were determined in whole cell lysates. The strains TEA028-*rne*, TEA028-*rne*-Δ*rng*, and TEA028-*rne*-Δ*rng* (p*rng*) underproduce RNase E when the medium is supplemented with 0.1 µM IPTG. Means and standard errors of at least 4 biological replicates are graphed. Abbreviations: *rng*: TEA028-Δ*rng*; *rne*: TEA028-*rne*; *rne rng*: TEA028-*rne*-Δ*rng*; *rne rng* (p*rng*): TEA028-*rne*-Δ*rng* (p*rng*). * Adjusted *P* value versus TEA028 = 0.0001; ** adjusted *P* value versus TEA028 < 0.0001; # adjusted *P* value versus TEA028-*rne* at 10 h = 0.0001; & adjusted *P* value versus TEA028-*rne* at 10 h = 0.0024.

Next, Stx2 production was measured in extracts prepared from MMC-treated cultures of TEA028 and its RNase E and RNase G derivatives. Specifically, the EHEC strain TEA028 and its derivatives produce Stx2a subtype. At 6 h after MMC addition, lysates from the strains lacking RNase G (TEA028-Δ*rng*) or underproducing RNase E (TEA028-*rne* at 0.1 µM IPTG) had toxin levels that were not significantly different from TEA028 (control) (Fig. 3). Similarly, strain TEA028-*rne* at 100 µM IPTG (normal RNase E levels) produces toxin as the control (Supplementary Fig. S4, and Ref.^49^). Deficiency of RNase E and G (TEA028-*rne*-Δ*rng* at 0.1 µM IPTG) resulted in 245 ± 36 ng/mg of protein (mean ± standard error) contrasting with the production of 4,677 ± 173 ng/mg of protein (mean ± standard error) by TEA028. Because of the slower kinetics of growth and lysis in TEA028-*rne*-Δ*rng* at 0.1 µM IPTG, Stx2 was also measured in cell extracts at later time points (10 and 24 h) after MMC addition. Toxin production by TEA028 was not measured beyond the 6-h period because at this point cells are mostly lysed, and thus, a small increment or no increment in Stx2 accumulation would be expected at later time points. In cultures of TEA028-*rne*-Δ*rng* at 0.1 µM IPTG, toxin accumulation increased 1.7-fold at 10 h and 2.2-fold at 24 h when compared with the 6-h time point. However, there was still about eightfold difference in the mean of Stx2 levels between cell lysates of the control (TEA028) at 6 h and cell lysates of TEA028-*rne*-Δ*rng* at 0.1 µM IPTG at 24 h. For comparison, Stx2 concentrations under RNase E scarcity alone (TEA028-*rne* at 0.1 µM IPTG) were still high at 10 h. Complementation of the *rng* deletion in the TEA028-*rne*-Δ*rng* (p*rng*) strain, partially restored Stx2 production at 6, 10, and 24 h after MMC addition. Partial complementation in the production of toxin and phages may be caused by higher than normal physiological levels of RNase G produced in the complemented strain. To examine this possibility, RNase G concentrations were analyzed by Western blotting in TEA028, its *rng* deletion mutant derivatives, and in an *E. coli* laboratory strain overexpressing RNase G as a positive control. Supplementary Fig. S5 shows RNase G was detected as a prominent band of the expected molecular weight in the positive control. However, RNase G was undetectable in the parental strain TEA028, but it was detected in the complemented strain. Attempts to improve the sensitivity of detection in TEA028 failed. Therefore, RNase G in EHEC is at low physiological levels in accordance with previous data in nonpathogenic *E. coli*^51^. As a strategy to reduce RNase G concentrations in the complemented strain, the ATG start codon of the *rng* gene was mutated to GTG. In *E. coli*, the GTG codon reduces the efficiency of translation initiation and is a more infrequent start codon than ATG^52^. Complementation with the GTG-mutated *rng* gene resulted in higher toxin yields than complementation with the wild-type variant (Supplementary Fig. S6), suggesting that higher-than-normal levels of RNase G in the TEA028-*rne*-Δ*rng* (p*rng*) strain caused the partial complementation result.

### Quantification of mRNA levels of the *stx2*, *recA*, and *Q* mRNAs in EHEC and its RNases E and G derivatives

Reverse transcription real-time PCR (RT-qPCR) was used to examine whether the reduction in Stx2 levels under RNases E and G deficiency could be explained by a concomitant reduction in *stx2* mRNA levels. In addition, *recA* and *Q* transcript levels were also examined since expression of these genes is essential for initiation of the *stx2* phage lytic cycle and Stx2 production when DNA damage occurs. Cultures of each strain were grown to OD_600_ of 0.3–0.35 (time 0) at which point MMC (1 µg/mL) was added; thereafter, aliquots were collected at various time points for total RNA extraction and RT-qPCR analysis. For each gene, fold change was calculated as the cDNA present at each time point with respect to the cDNA at time 0 (described in “Methods” section).

**Figure 4:**
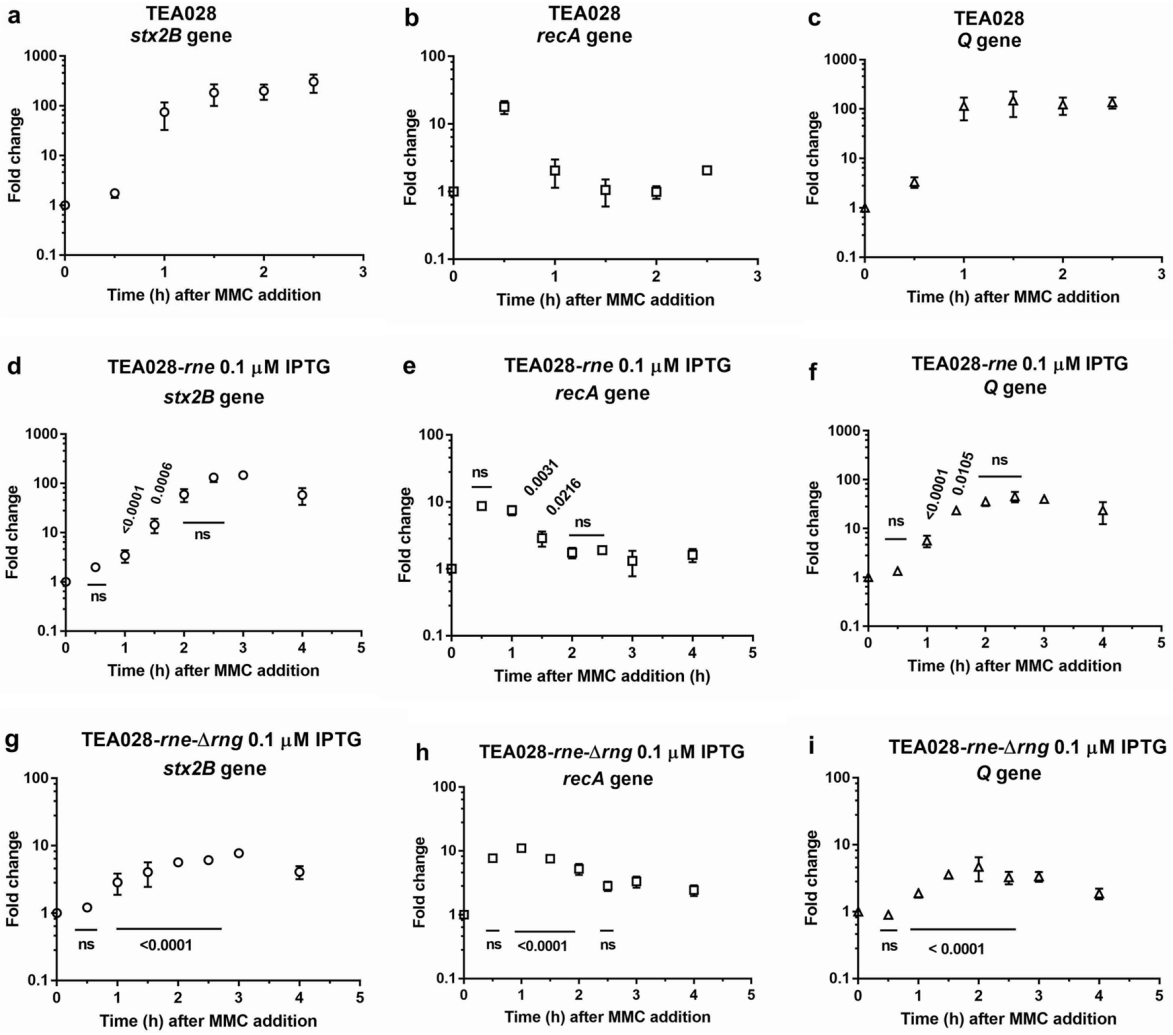
Kinetics of mRNA concentrations of *stx2B*, *recA*, and *Q* genes in EHEC TEA028 and its RNases E and G derivatives. Cultures of TEA028 (parental strain) and its RNase E/G derivatives were grown in Luria Bertani medium to an optical density at 600 nm of 0.30–0.35 (time 0) at which point the cultures were treated with MMC (1 µg/mL) to induce the phage lytic cycle and toxin production. Thereafter, samples were collected at the indicated time intervals for total RNA extraction and reverse transcription real-time PCR. The strains TEA028-*rne* and TEA028-*rne*-Δ*rng* underproduce RNase E when the medium is supplemented with 0.1 µM IPTG. The concentration of cDNA molecules for each gene was normalized to the concentration of cDNA molecules of 16S rRNA. Fold change was calculated as the ratio of the normalized cDNA molecules at the indicated time points after MMC addition to the normalized cDNA molecules before the addition of MMC (time 0). Adjusted *P* values versus TEA028 (control) are indicated in each graph, *ns* not significant. Means and standard errors of 3–4 biological replicates are shown. (**a**) TEA028, *stx2B* gene. (**b**) TEA028, *recA* gene. (**c**) TEA028, *Q* gene. (**d**) TEA028-*rne* 0.1 µM IPTG, *stx2B* gene. (**e**) TEA028-*rne* 0.1 µM IPTG, *recA* gene (**f**) TEA028-*rne* 0.1 µM IPTG, Q gene. (**g**) TEA028-*rne*-Δ*rng* 0.1 µM IPTG, *stx2B* gene. (**h**) TEA028-*rne*-Δ*rng* 0.1 µM IPTG, *recA* gene. (**i**) TEA028-*rne*-Δ*rng* 0.1 µM IPTG, *Q* gene.

In the TEA028 strain, the *stx2B* transcript reached high levels 1 h after the addition of MMC (Fig. 4a). Similar *stx2B* mRNA kinetics were observed in strains TEA028-*rne* at 100 µM IPTG (Supplementary Fig. S7b) and TEA028-Δ*rng* (Supplementary Fig. S7c). Under RNase E deficiency, high transcript levels were observed at 2.5 h and later time intervals, after an initial delay (Fig. 4d). In contrast, deficiency of both RNases reduced significantly the levels of *stx2* transcripts, with 50-fold decrease at 2.5 h when compared with the control (TEA028) (Fig. 4a, g).

The *recA* transcripts showed similar kinetics in the TEA028, TEA028-*rne* at 100 µM IPTG, and TEA028-Δ*rng* strains, with high levels at 30 min and falling afterwards to a fold change below 1 in some experiments (Supplementary Fig. S7a, S7b, and S7c). Depletion of RNase E or of RNases E and G did not reduce *recA* transcript levels, and fold changes were above 1 at all time points in most of the experiments (Fig. 4e,h). In particular, *recA* transcript levels stayed significantly elevated at three time points under RNases E and G scarcity when compared with the control (TEA028) (Fig. 4b,h).

The kinetics of *Q* transcript levels were similar in TEA028, TEA028-*rne* at 100 µM IPTG, and TEA028-Δ*rng* (Supplementary Fig. S7a, S7b, and S7c), resembling the expression pattern of *stx2* mRNA. Under RNase E scarcity, there was a slight delay in the *Q* mRNA peak, but the levels were as high as in the parental strain at later time points (Fig. 4f). Under RNases E and G deficiency, the *Q* transcript levels were significantly lower at all time points with about 42-fold reduction at 2.5 h when compared with TEA028 (Fig. 4c,i). These findings indicate that RNases E and G scarcity significantly impaired Q expression, which could have contributed to reduction in *stx2* transcription and Stx2 production.

### Determination generation times in EHEC and its RNases E and G derivative strains

In addition to the reduction in *Q* transcript levels, the impairment of Stx2 production under RNases E and G deficiency may be an indirect consequence of increased generation times from the burden of slowed RNA metabolism. In support of this hypothesis, the generation time was significantly increased in TEA028-*rne*-Δ*rng* at 0.1 µM IPTG when compared with the control or deficiency of RNase E alone (Supplementary Fig. S8).

## Discussion

This study establishes that depletion of RNases E and G significantly impair the production of both Stx2 and *stx2-*phages in EHEC. In contrast, the absence of RNase G does not impair the production of either toxin or phages, and deficiency of RNase E impairs phage production only (this study and Ref.^50^). The inducing agent used in this study, MMC, strongly triggers the cellular response to DNA damage. Thus, cells deficient in both RNases still undergo the initiation of the phage lytic cycle with concomitant toxin production although at a slower rate and resulting in lower Stx2 yields even at 24 h after MMC addition. RecA and Q proteins are critical for the initiation of the phage lytic cycle and toxin production. The reduced *stx2* transcript levels under RNase E and G deficiency cannot be explained by a change in *recA* transcript levels, which remained higher than in the control. In contrast, RNase E and G scarcity caused a drastic reduction in transcript levels of the Q antiterminator, which is required for transcription to continue to *stx2* genes. This result can explain, at least partially, the observed decrease in *stx2* transcript concentrations. In addition, the impaired expression of Q could have affected the transcription of late genes required for the assembly of phage particles. The reduction of Q transcript levels could be the result of impaired *Q* gene transcription or transcript stability. If the latter scenario is correct, RNases E/G effect on *Q* mRNA lifetimes is probably indirect because they are required for *Q* transcripts to reach high levels.

Deficiency of both RNases likely creates metabolic burden by affecting the stability and processing of many RNAs. As a consequence, the growth rate is decreased, probably having an indirect effect on the production and release of toxin and phages. The mechanisms underlying these effects are probably many and complex since depletion of RNase E alone changes the half-life of thousands of transcripts in nonpathogenic *E. coli*^53^. In EHEC, direct RNase E or G degradation or processing of transcripts encoding virulence factors, such as Shiga toxin, has been scarcely explored^37,38,54^.

The importance of the production of actual phage particles in disease progression is debatable. Some studies indicate that infection of commensal bacteria by *stx* phages may amplify the production of toxin^55,56^, potentially worsening disease outcomes. However, subsequent work with a microbiome-replete murine model of EHEC infections indicates that the actual production of phage particles is negligible for disease^57^. Nevertheless, potential transmission of *stx* genes to naive bacterial hosts is problematic, as exemplified by the novel and highly virulent Stx2-producing *E. coli* serotype O104:H4 that caused an outbreak in Europe in 2011^58^. Therefore, the reduction of both toxin and infective phage particles produced under RNases E and G scarcity is important.

Despite remarkable advances in the understanding of EHEC pathogenesis and its virulence factors, specific therapeutics against EHEC infections are still lacking. The neutralization of Stx by a binding agent (Synsorb P) was ineffective in a multicenter randomized controlled clinical trial, and the authors concluded that success of similar strategies was doubtful^59^. Although humanized monoclonal antibodies against Stx1 or Stx2 showed efficacy at controlling fatal complications in animal models^60–63^, clinical trials have not advanced beyond the determination of safety and pharmacokinetic parameters^63–65^. In the United States, antibiotics are contraindicated for treatment of Stx-producing *E. coli* infections^28^.This topic remains controversial since there are some conflicting data from in vitro, animal model and clinical studies (reviewed in^66^). Nevertheless, it is clear that the effect of antibiotics on Stx production depends on the antimicrobial compound and the particular Stx-producing *E. coli* strain. Given the complexity of the problem, the therapeutic potential of the findings reported here is unknown, at this moment. Inhibitors of purified RNase E/G have been isolated^67,68^; however, there are currently no reported inhibitors of those enzymes that can be tested in vivo, i.e., on the EHEC capacity to produce toxin.

## Methods

### Strains and culture conditions

Supplementary Table S1 describes the strains used in this study. For each experiment, the strains were freshly streaked on plates directly from glycerol stocks maintained at − 70 °C. To prepare the inoculum, one colony was inoculated into Luria Bertani (LB) medium and incubated for 12–13 h at 37 °C with agitation (130 rpm). The cells were collected by centrifugation, resuspended in phosphate buffered saline or LB, and inoculated into LB medium at a ratio 1:500. The cultures were incubated at 37 °C with agitation to OD_600_ of 0.30–0.35, at which point MMC (1 µg/mL) was added; thereafter, samples were collected at various time intervals. The LB medium was supplemented as follows: tetracycline (tet) (6 µg/mL) in the case of TEA028 and TEA028-Δ*rng* strains; and tet (6 µg/mL), ampicillin (amp) (100 µg/mL), chloramphenicol (25 µg/mL), and isopropyl β-D-1-thiogalactopyranoside (IPTG) (100 µM or 0.1 µM) in the case of TEA028-*rne*, TEA028-*rne*-Δ*rng*, TEA028-*rne*-Δ*rng* (p*rng*), and TEA028-*rne*-Δ*rng* (pGTG) strains.

### Oligonucleotides

The oligonucleotide sequences used in this study are described in Supplementary Table S2.

### Construction of TEA028-*Δrng* and TEA028-*rne*-*Δrng* strains

The *rng* gene from strainsTEA028 and TEA028-*rne* was deleted following the gene-doctoring procedure^69^. The kanamycin (kan) resistance gene was amplified with primers f-EcoRI-rng and r-XhoI-rng (Supplementary Table S2) using plasmid pDOC-K as the template. The PCR fragment was cloned between the EcoRI and XhoI sites of plasmid pDOC-C (or pDOC-C-Gen [see below]), which carries an amp resistance marker, and the *sacB* gene that confers resistance to sucrose. The resulting plasmid was then introduced into the strain TEA028. Next, TEA028 was transformed with the plasmid pACBSCE, which carries the genes encoding for the λ-Red proteins and the gene encoding for the restriction enzyme I-SceI. Cultures were grown for 2 h at 37 °C, and then the cells were pelleted and resuspended in LB supplemented with 0.5% L-arabinose to induce the expression of λ-Red proteins. Appropriate dilutions were plated to select for kan resistant and sucrose insensitive recombinants. The colonies were also checked for loss of the donor plasmids. Next, the recombinants were screened with PCR primers CC1 and CC2 and with complementary primers to the *rng* gene flanking region as described by Lee et al.^69^. To eliminate the kan resistance marker inserted into the *rng* gene, the Flp recombinase was produced from the pCP20 plasmid (*E. coli* Genetic Stock Center), which was then eliminated by incubation at the restrictive temperature. To delete the *rng* gene in the TEA028-*rne* strain, which is amp resistant, the pDOC-C plasmid was modified by introducing a gentamicin resistance gene (*aacC1*). The *aacC1* gene was amplified with PCR primers f-SphI-gen and r-SphI-gen (Supplementary Table S2) from plasmid pBAMD1-6 (Addgene). The PCR fragment was cloned into the SphI restriction site of plasmid pDOC-C to generate plasmid pDOC-C-Gen. To delete the *rng* gene from the TEA028-*rne* strain, the same procedure described above was followed, except the LB medium was supplemented with 100 µM IPTG to induce production of RNase E. After the *rng* gene was deleted, we introduced into the TEA-*rne*-Δ*rng* strain the plasmid pLacI^Q^ which carries the *lacI*^Q^ gene or the plasmid p*rng* which carries the *lacI*^Q^ gene and the *rng* gene. The LB plates were supplemented with antibiotics as described by Lee et al.^69^ and gentamicin at 10 µg/mL, when appropriate.

### Construction of plasmid p*rng* and site-directed mutagenesis

The *rng* gene was amplified using genomic DNA as template and primers f-XbaI-rng and r-HindIII-term-rng. The M13 transcriptional terminator, which has high termination efficiency, was added at the end of the primer r-HindIII-term-rng^70^. The *rng* sequence was cloned into XbaI and HindIII restriction sites of plasmid pLacI^Q^^49^ and confirmed by Sanger sequencing (Genewiz, Inc.). The resultant plasmid (p*rng*) was introduced into the EHEC strain TEA028-*rne*-Δ*rng*. The Q5 Site-Directed Mutagenesis Kit (New England Biolabs) was used to change the ATG start codon to GTG of the cloned *rng* gene into plasmid p*rng*. The change in the *rng* sequence was confirmed by Sanger sequencing.

### Shiga toxin quantification

Stx2 was quantified in whole cell extracts by the receptor ELISA technique (RELISA), as described in Supplementary Information.

### Plaque assays

Culture supernatants were collected after centrifugation at 15,000 × g for 5 min, treated with one-tenth volume of chloroform, and then mixed with an equal volume of 4 M (NH_4_)_2_SO_4_ solution before storage at 4 °C. Phages were quantified by the double agar overlay method or the drop method as described by Thuraisamy and Lodato^50^. Plaque numbers were log-transformed before statistical analysis.

### RNA isolation and mRNA quantification

Culture samples (3, 5, or 6 mL) were collected at 4 °C and added in a 10:1 ratio to a mixture of 95% ethanol and 5% saturated phenol. After centrifugation, the cell pellets were stored at − 70 °C. To extract total RNA, the cell pellets were resuspended in 300 µL of lysis buffer (10 mM Tris-Cl, pH 8.0, 1 mM EDTA, 0.5 mg/mL lysozyme), 20 µL of 20% SDS, and 20 µL of water. The lysate was incubated at 64 °C for 2 min, and then 1.2 mL of TRI Reagent solution (Invitrogen) was added. Next, total RNA was isolated with the Direct-zol RNA Miniprep kit (Zymo Research). On-column DNA digestion was performed during the RNA extraction following the manufacturer’s directions. RNA was eluted in water, and the concentration determined with a NanoDrop 2000c spectrophotometer. To digest remaining DNA contamination, an aliquot of each RNA sample was treated with TURBO DNase (Ambion) in a reaction mixture containing SUPERase∙In RNase Inhibitor (Thermo Scientific). The total RNA was then repurified using saturated phenol and chloroform following standard procedures and stored at − 70 °C. The RNA integrity was checked by agarose gel electrophoresis, and potential DNA contamination was detected by qPCR amplification with primers f-qPCR-stx2B and r-qPCR-stx2B (Supplementary Table S2). cDNA was synthesized with the iScript cDNA Synthesis Kit (Bio-Rad) following the manufacturer’s instructions. The *stx2B*, *recA*, *Q*, and *rrsH* genes were amplified by qPCR from appropriate dilutions of cDNA using the primers described in Supplementary Table S2. The reactions (10 µL) were performed in a CFX Connect real-time detection system (Bio-Rad) using the iTaq Universal SYBR Green Supermix (Bio-Rad). The cycling protocol was 95 °C for 3 min and then 40 cycles at 95 °C for 5 s and 60 °C for 30 s. A melting curve analysis was completed afterwards to confirm the presence of only one product of amplification and the absence of primer dimers. The initial concentration of molecules (N_0_) was calculated using the open-source software LinReg^71,72^. Then, the N_0_ of *stx2B*, *recA*, or *Q* cDNA was normalized to the N_0_ of the *rrsH* cDNA for each experiment. For each gene, the fold change was calculated as the ratio of the normalized N_0_ at each time point after MMC addition to the normalized N_0_ before the MMC addition (time 0). Fold changes were log-transformed before statistical analysis.

### Statistical analysis

The results of this study are derived from at least three biological replicates. The statistical analysis was performed with the software GraphPad Prism 7.0. Groups were compared using one-way or two-way analysis of variance and Dunnett’s or Sidak’s multiple comparison test, or a t-test when two groups were compared. Measurements of toxin, protein concentrations, and qPCR were performed in three technical replicates.

## Supplementary Information

Supplementary Information.

## Data Availability

Data are available from the author upon request.
