# Supplementary material for: The effect of two ribonucleases on the production of Shiga toxin and stx-bearing bacteriophages in Enterohaemorrhagic Escherichia coli
Source: Sci Rep. 2021 Sep 15;11:18372. doi: 10.1038/s41598-021-97736-z (PMC8443680; doi:10.1038/s41598-021-97736-z)
Supplement: Supplementary file 1 — Supplementary Information. [file 41598_2021_97736_MOESM1_ESM.pdf]

Supplementary Information

The Effect of Two Ribonucleases on the Production of Shiga Toxin and *stx*-Bearing Bacteriophages in Enterohaemorrhagic *Escherichia coli*

Patricia B. Lodato*

A.T. Still University, Kirksville College of Osteopathic Medicine, Department of Microbiology and Immunology, Kirksville, Missouri, 63501, USA.
patricialodato@atsu.edu
*Correspondence to: Patricia B. Lodato

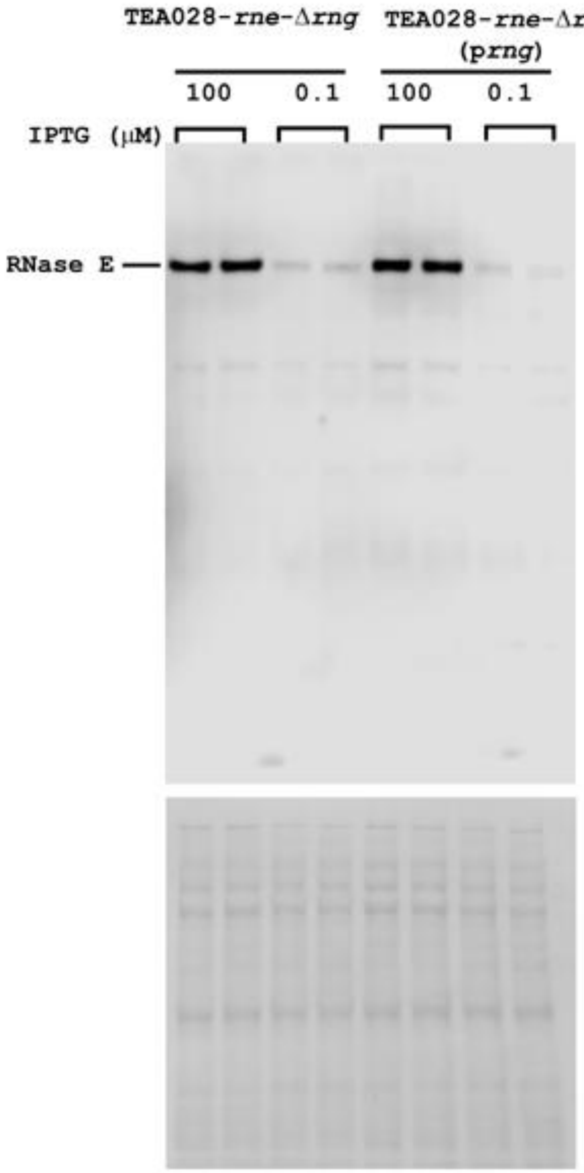

Supplementary Figure S1. Production of RNase E is dependent on isopropyl β-D-1-thiogalactopyranoside (IPTG) in the EHEC strains TEA028-*rne*-Δ*rng* and TEA028-*rne*-Δ*rng* (p*rng*). In both strains, the RNase G-encoding gene (*rng*) is deleted, and transcription of the RNase E-encoding gene (*rne*) is IPTG-inducible. The *rng* gene is expressed from a plasmid in the complemented strain (TEA028-*rne*-Δ*rng* (p*rng*)). For each strain, two independent bacterial cultures were grown in Luria Bertani medium supplemented with IPTG as indicated. Cells were collected from cultures grown to optical density at 600 nm of 0.30-0.35, and cell extracts were prepared as described in the Methods section. Cellular proteins (20 µg) were subjected to SDS-polyacrylamide gel electrophoresis, and RNase E was detected by immunoblotting using anti-RNase E antibodies (upper panel). Before immunodetection, the blot was stained with Ponceau S to show the total protein loaded in each lane (lower panel).

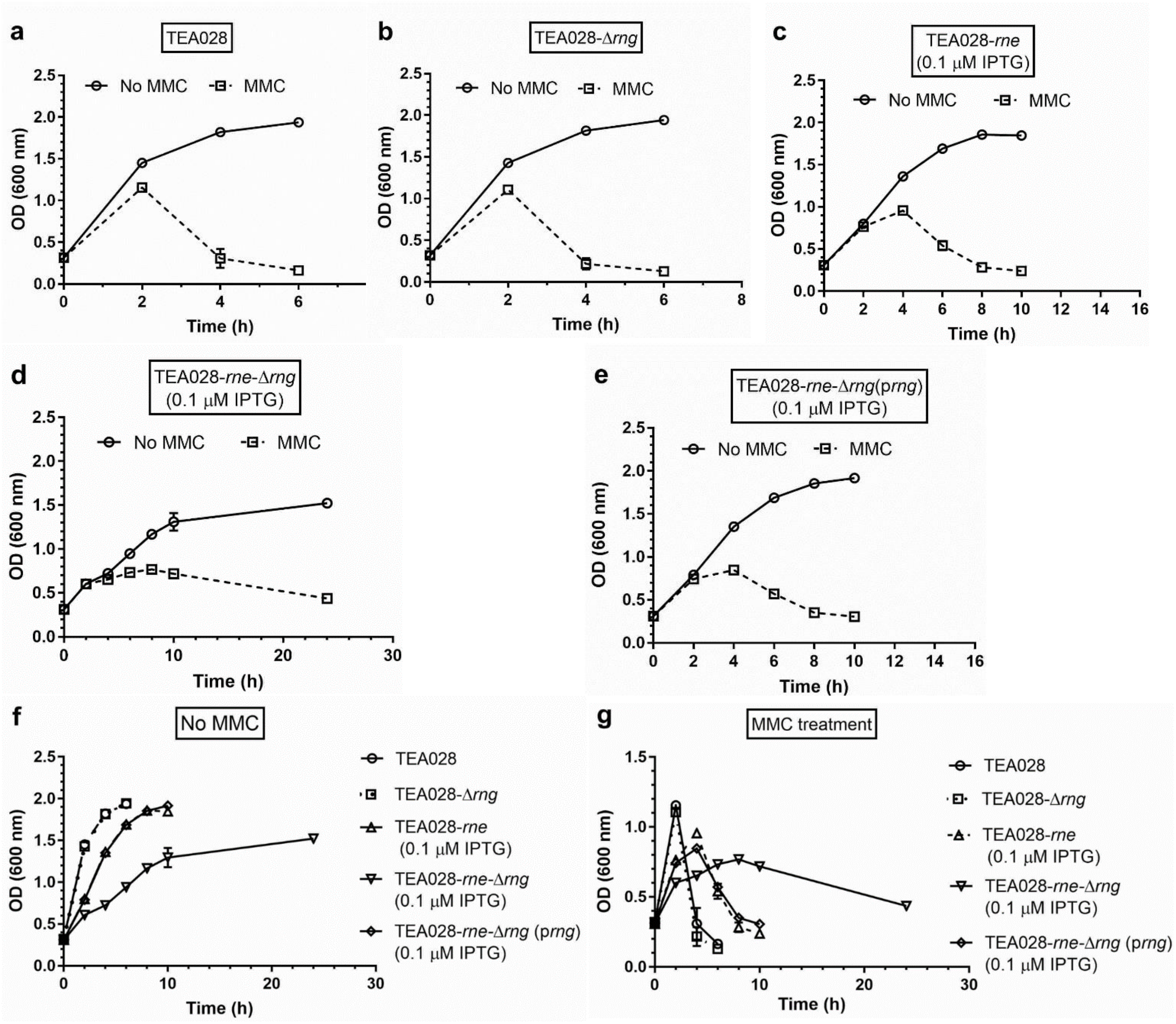

Supplementary Figure S2. Growth of EHEC TEA028 and its RNases E and G derivative strains in cultures treated or non-treated with mitomycin C (MMC). Cultures of TEA028 (parental strain) and its RNases E and G derivatives were grown in Luria Bertani medium to optical density at 600 nm (OD_{600}) of 0.30-0.35 (time 0) at which point the cultures were split and an aliquot was treated with MMC (1 µg/mL) to induce the *stx2* phage lytic cycle. Thereafter, samples were collected at various time points to measure OD_{600}. The strains TEA028-*rne*, TEA028-*rne*-Δ*rng*, and TEA028-*rne*-Δ*rng* (p*rng*) underproduce RNase E when the medium is supplemented with low levels of isopropyl β-D-1-thiogalactopyranoside (IPTG), as indicated. Means and standard errors of at least 4 biological replicates are graphed. Lack of error bars indicates that the standard error was smaller than the plot symbol. (**a**) TEA028. (**b**) TEA028-Δ*rng*. (**c**) TEA028-*rne*. (**d**) TEA028-*rne*-Δ*rng.* (**e**) TEA028-*rne*-Δ*rng*(p*rng*). (**f**) Growth of EHEC strains, as indicated, in medium without MMC. (**g**) Effect of MMC treatment on the growth of EHEC strains, as indicated.

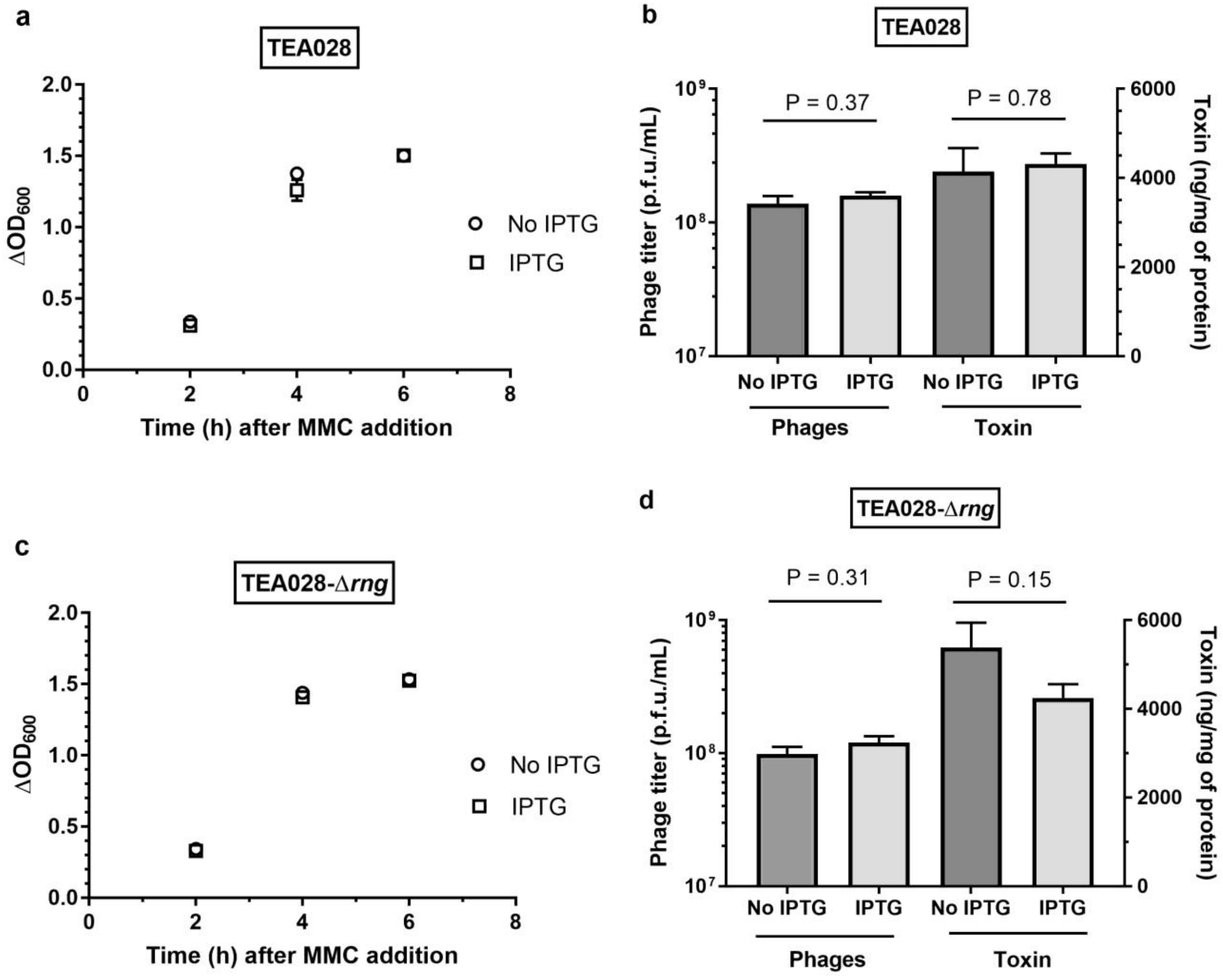

Supplementary Figure S3. Effect of isopropyl β-D-1-thiogalactopyranoside (IPTG) on the response of EHEC TEA028 and its derivative strain TEA028-Δ*rng* to mitomycin C (MMC) treatment. Cultures in Luria Bertani medium without IPTG or containing 100 µM IPTG were grown to optical density at 600 nm (OD_{600}) of 0.30-0.35. At this point, MMC (1 µg/mL) was added to a fraction of each culture to induce the phage lytic cycle. Thereafter, growth and cell lysis were followed by measuring OD_{600} at the indicated time intervals. (**a**, **c**) Difference in OD_{600} measurements between non-treated and MMC-treated cultures. (**b**, **d**) Plaque forming units in culture supernatants and toxin (Stx2a subtype) concentrations in culture lysates were determined at 6 h after MMC addition. The means and standard errors of three independent cultures for each condition are shown. P values are indicated on the graphs. Some error bars are smaller than the plot symbols.

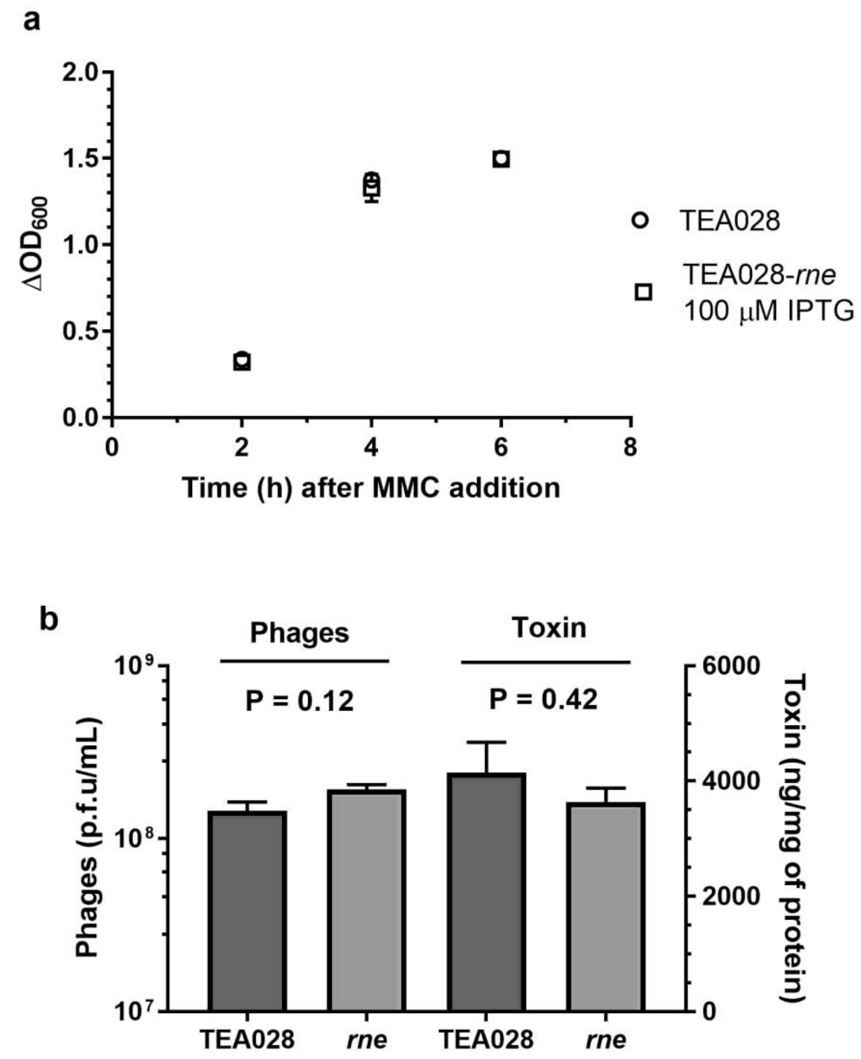

Supplementary Figure S4. Response to mitomycin C (MMC) treatment of EHEC TEA028 and its derivative TEA028-*rne* strain producing normal levels of RNase E.

Cultures in Luria Bertani medium were grown to optical density at 600 nm (OD_{600}) of 0.30-0.35. At this point, MMC (1 µg/mL) was added to a fraction of each culture to induce the phage lytic cycle. Thereafter, growth and cell lysis were followed by measuring OD_{600} at the indicated time intervals. The production of physiologic RNase E levels in TEA028-*rne* was induced with 100 µM IPTG. (**a**) Difference in OD_{600} measurements between non-treated and MMC-treated cultures. (**b**) Plaque forming units in culture supernatants and toxin (Stx2a subtype) concentrations in culture lysates were determined at 6 h after MMC addition. The means and standard errors of three independent cultures for each condition are shown. P values are indicated in the graph.

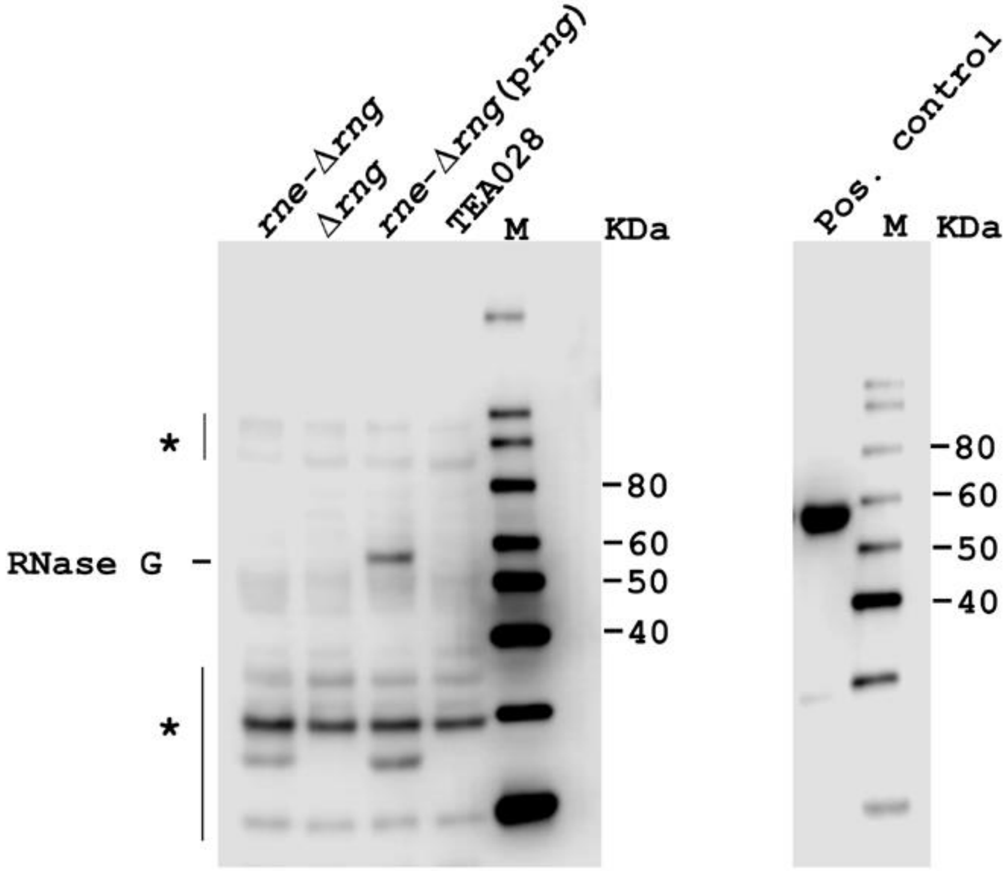

Supplementary Figure S5. Production of RNase G in the parental strain TEA028 and its RNases E and G derivatives. EHEC TEA028, TEA028-Δ*rng*, TEA028-*rne*-Δ*rng*, and TEA028-*rne*-Δ*rng* (p*rng*) were grown overnight at 37°C in Luria Bertani medium, which was supplemented with 100 µM IPTG in the case of the latter two strains. Cells were pelleted, and lysates were prepared as described in Methods. Proteins (left panel, 30 µg; right panel, 10 µg) were separated by sodium dodecyl sulfate polyacrylamide gel electrophoresis, and RNase G was detected by immunoblotting with anti-RNase G antibodies. The positive control was an *E. coli* K-12 strain overexpressing RNase G from an IPTG-inducible promoter [1]. The asterisks point to unspecific crossreacting proteins with the RNase G antiserum. The two blots were prepared on different days.

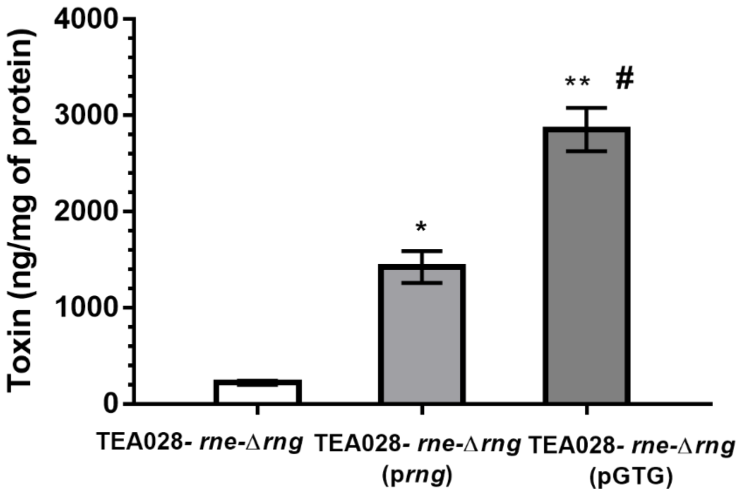

Supplementary Figure S6. Production of toxin (Stx2a subtype) by EHEC with deficiency in RNases E and G and in the RNase G complemented strains. Cultures of the EHEC strains were grown in Luria Bertani medium supplemented with 0.1 µM isopropyl β-D-1-thiogalactopyranoside (IPTG) to an optical density at 600 nm of 0.30-0.35 at which point the cultures were treated with MMC (1 µg/mL) to induce the phage lytic cycle and toxin production. Samples were collected at 6 hours, and stored at −80°C until toxin concentration was measured in whole lysates as described in Methods. The plasmid p*rng* carries the *rng* gene transcribed from its own promoter; in the plasmid pGTG, the ATG start codon of *rng* was changed to GTG to reduce translation initiation of *rng*. The mean and standard error of 4-6 biological replicates are graphed. * adjusted P value versus TEA028-*rne*-Δ*rng* = 0.0006; **adjusted P value versus TEA028-*rne*-Δ*rng* = 0.0001; # adjusted P value versus TEA028-*rne*-Δ*rng* (p*rng*) = 0.0018.

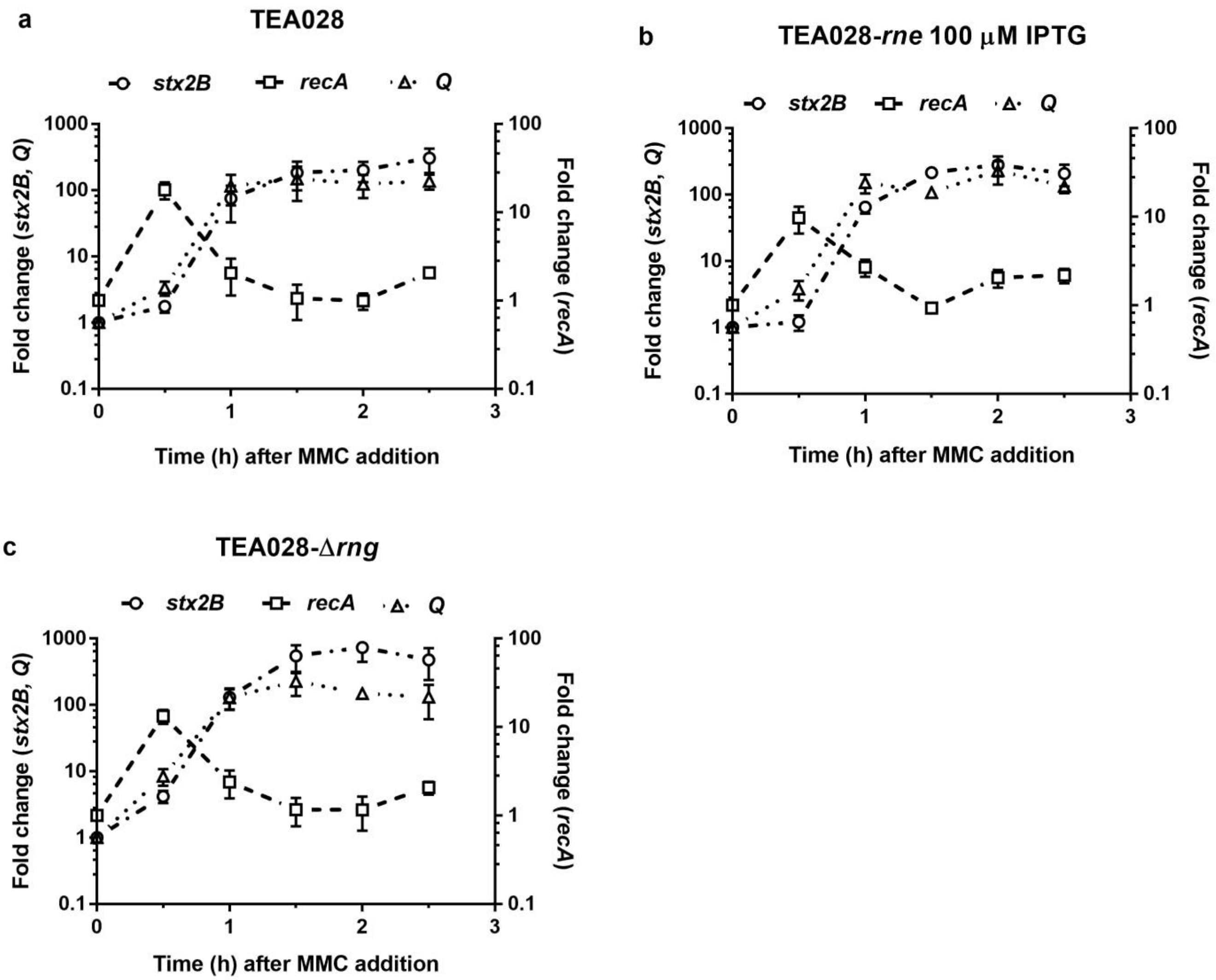

Supplementary Figure S7. Kinetics of mRNA concentrations of *stx2B*, *recA*, and *Q* genes in EHEC TEA028, TEA028-*rne*, and TEA028-*Δrng*. Cultures of TEA028 (parental strain) and its RNase E/G derivatives were grown in Luria Bertani medium to an optical density at 600 nm of 0.30-0.35 (time 0) at which point the cultures were treated with MMC (1 µg/mL) to induce the phage lytic cycle and toxin production. Thereafter, samples were collected at the indicated time intervals for total RNA extraction and reverse transcription real-time PCR. TEA028-*rne* produces normal levels of RNase E when supplemented with 100 µM IPTG. The concentration of cDNA molecules for each gene was normalized to the concentration of cDNA molecules of 16S rRNA. Fold change was calculated as the ratio of the normalized cDNA molecules at the indicated time points after MMC addition to the normalized cDNA molecules before the addition of MMC (time 0). (**a**) TEA028. (**b**) TEA028-*rne* at 100 µM IPTG. (**c**) TEA028-Δ*rng*. The mean and standard error of three biological replicates are shown. The fold changes of *stx2B*, *recA* and *Q* genes of TEA028 versus TEA028-*rne* at 100 µM IPTG or TEA028-Δ*rng* were not significantly different.

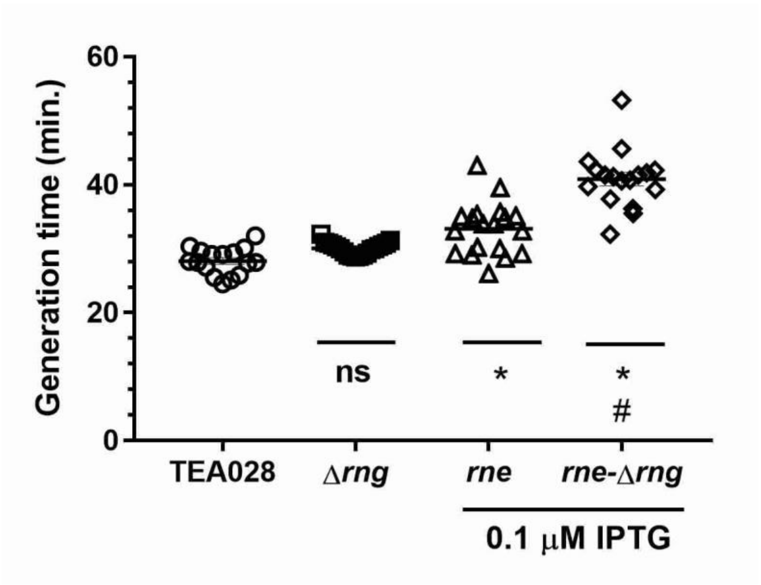

Supplementary Figure S8. Growth rate of EHEC TEA028 and its RNases E and G derivative strains. The strains were grown in Luria Bertani medium in a multi-well plate and incubated at 37°C in a plate reader. The medium was supplemented with antibiotics and 0.1 µM isopropyl β-D-1-thiogalactopyranoside (IPTG) as indicated. Growth was followed by measuring optical density at 600 nm every 15-20 minutes. In the graph: TEA028 (parental strain), Δ*rng*=TEA028-Δ*rng*, *rne*=TEA028-*rne*, and *rne*-Δ*rng*=TEA028-*rne*-Δ*rng*. Each data point represents the calculation of generation time from one growth curve. * adjusted P value versus TEA028 = 0.0001; # adjusted P value versus TEA028-*rne* < 0.0001; ns= not significant versus TEA028.

Supplementary Table S1. Strains used in this study

Strain	Description	Source or reference
TEA028	Derivative of EHEC serotype O157:H7 strain EDL933, Δ*galETKM::tetA.*	[2]
TEA028-*rne*	Derivative of TEA028 which carries the IPTG*-inducible, HA- and His_6-tagged *rne* gene. This strain also produces the $LacI^Q$ repressor from a plasmid.	[3]
TEA028-Δ*rng*	TEA028 strain carrying a deletion of the RNase G-encoding gene (*rng*).	This study
TEA028-*rne*-Δ*rng*	Derivative of TEA028 which carries the IPTG-inducible, HA- and His_6-tagged *rne* gene and a deletion of the *rng* gene. This strain also produces the $LacI^Q$ repressor from a plasmid.	This study
TEA028-*rne*-Δ*rng* (p*rng*)	TEA028-*rne*-Δ*rng* expressing RNase G from its own promoter, cloned in a plasmid that also produces the $LacI^Q$ repressor.	This study
TEA028-*rne*-Δ*rng* (pGTG)	ATG to GTG change in the start codon of the *rng* gene which is cloned in the same plasmid carrying the *lacI*^{Q} gene.	This study
MC1061	*Escherichia coli* strain used as a host to determine phage titer.	[4]

***** IPTG: isopropyl β-D-1-thiogalactopyranoside

Supplementary Table S2. Oligonucleotides used in this study

Name/orientation	Sequence (5' to 3')	Source or reference
f-EcoRI-rng/forward	ccggaattc*gctgaattgttagtaaacgtaacgccttcggaaacgcgagtggcgtatatt*gaccggtcaattggctggag [a]	This study
r-XhoI-rng/reverse	ccgctcgag*cattacgacgtcaaactgctcctggttatagagcggttcaatttgtactttaa*aatatcctccttagttcc [b]	This study
CC1/reverse	cttaagttctacgtgttccgc	[5]
CC2/forward	catgctggagttcttcgccc	[5]
f-SphI-gen/forward	acatgcatgcgaaccttgaccgaacgcagc [c]	This study
r-SphI-gen/reverse	acatgcatgcaggagtccaagactagtctgtctc [c]	This study
f-XbaI-rng/forward	acattctagaattctcgattgcctggtgg [d]	This study
r-HindIII-term-rng	acagaagctt*aaaaaaaaaggctccaaaaggag*cccgtcactccttgtctgctaa [e]	This study
f-qPCR-stx2B	gatgtttatggcggttttatttgc	[6]
r-qPCR-stx2B	tggaaaactcaattttacctttagca	[6]
f-qPCR-RecAa	aaccatctctaccggttcgc	This study
r-qPCR-RecAa	cggaagattccggtccgtag	This study
f-qPCR-Qa	gactgatccccgaaaaagta	[7]
r-qPCR-Qa	caaccagcaagtcatgcag	[7]
f-qPCR-16Sd	cgtgttgtgaaatgttgggttaa	This study
r-qPCR-16Sd	ccgctggcaacaaaggataa	This study

a. An EcoRI restriction site is underlined; the *rng* sequence is in italics.
b. A XhoI restriction site is underlined; the *rng* sequence is in italics.
c. A SphI restriction site is underlined.
d. A XbaI restriction site is underlined.
e. A HindIII restriction site is underlined; the sequence in italics corresponds to the M13 transcriptional terminator.

Methods

Preparation of protein extracts and Western blotting

Cells from bacterial cultures were collected by centrifugation (15,000 × g for 5 min), resuspended in water, and mixed with Bacterial Protein Extraction Reagent (Thermo Fisher Scientific) in a 1:1 ratio. For immunoblotting detection of RNase E, the extract was then mixed with 4 M urea (final concentration). For detection of RNase G, the extract was disrupted with glass beads at 3,000 rpm for 2 minutes, and then the lysate was centrifuged at 15,000 × g for 5 minutes and the supernatant collected. Total protein was quantified by the DC Protein Assay kit (Bio-Rad). Cell extracts containing the same amount of total protein were mixed with Laemmli buffer (Bio-Rad) containing β-mercaptoethanol (5%), heated for 2 minutes at 85°C (for RNase G detection), and run in 10% or 4-20% SDS polyacrylamide gel electrophoresis. Proteins were transferred to a PVDF membrane (Immobilon-P, Millipore) by a semidry method. The membrane was blocked with chemiluminescent blocker (Immobilon Blok-CH) or I-Block solution (0.2% I-Block reagent [Thermo Fisher Scientific], 0.1% Tween 20) for 30 minutes and then incubated overnight with primary antibodies at 4°C. Washes were performed with phosphate-buffered saline containing 0.05% Tween 20 (PBST) (3 × 5 min), followed by incubation for 1.5-2 hours with HRP-conjugated anti-rabbit antibodies. After washing again, the membrane was incubated for 5 minutes with the chemiluminescent substrate (Clarity Western ECL substrate, Bio-Rad) and then scanned in a C-DiGit Blot Scanner (LiCor).

Shiga toxin quantification

Stx2 was quantified by the receptor ELISA technique (RELISA) as described by Thuraisamy and Lodato[8]. Briefly, the wells of Immunolon 1B 96-well plates (Thermo Fisher Scientific) were coated with 5 µg ceramide trihexosides (500 µg/mL, bottom spot) (Matreya). Then, the wells were filled with 100 µL of blocker (Immobilon Blok-CH), incubated for 2 hours, and washed twice with 100 µL of PBST. Next, 100 µL of appropriate dilutions in PBST of lysates or purified Stx2 (List Biological Laboratories) were dispensed in triplicates into the wells and incubated for 60-75 minutes. Lysates consist of culture samples mixed with Bacterial Protein Extraction Reagent in a 1:1 ratio and incubated for 5 minutes at 37°C. After washing three times with PBST, the wells were filled with 100 µL of rabbit anti-Stx2 antibodies (1:5,000 in blocker solution) (List Biological Laboratories), incubated for 2 hours, and then washed three times with PBST. Next, 100 µL of HRP-conjugated goat anti-rabbit IgG antibodies (1:5,000 diluted in blocker solution) (GeneTex) were added to the wells followed by 1-1.5 hours of incubation. The wells were washed twice with PBST and twice with PBS followed by the addition of 100 µL of substrate (1-Step Ultra TMB Blotting Solution, Thermo Fisher Scientific). After colour development (~15-20 min), the reactions were stopped with 100 µL of 2 M H_2SO_4. The coloured precipitate was dissolved by mixing in an orbital shaker, and then absorbance at 450 nm was measured in an Epoch 2 plate reader (BioTek). All incubations and washes were performed at room temperature. Standard curves were constructed with purified Stx2, and toxin concentrations were determined by interpolation of absorbance values.

Determination of generation times

Luria-Bertani medium was inoculated with overnight cultures of TEA028, or its derivatives, at a ratio 1:500 and dispensed into a multi-well plate. The multi-well plates were incubated at 37°C with agitation in a BioTek Epoch 2 plate reader, and cell growth was followed by measuring optical density at 600 nm at 15-20 minute intervals. Generation times were calculated as reported by Hall *et al*.[9], using the open source software GrowthRates 3.0. The generation time values were log-transformed before statistical analysis.

References

1. Richards, J. & Belasco, J.G. Distinct requirements for 5'-monophosphate-assisted RNA cleavage by *Escherichia coli* RNase E and RNase G. *J. Biol. Chem*. **291**, 5038-5048 (2016).

2. Ho, T. D. & Waldor, M. K. Enterohemorrhagic *Escherichia coli* O157:H7 *gal* mutants are sensitive to bacteriophage P1 and defective in intestinal colonization. *Infect. Immun*. **75**, 1661-1666 (2007).

3. Lodato, P. B, Thuraisamy, T., Richards, J. & Belasco, J. G. Effect of RNase E deficiency on translocon protein synthesis in an RNase E-inducible strain of enterohemorrhagic *Escherichia coli* O157:H7. *FEMS Microbiol. Lett*. **364**, fnx131. https://doi.org/10.1093/femsle/fnx131 (2017).

4. Casadaban, M. J. & Cohen, S. N. Analysis of gene control signals by DNA fusion and cloning in *Escherichia coli*. *J. Mol. Biol*. **138**, 179-207 (1980).

5. Lee, D. J. *et al*. Gene doctoring: a method for recombineering in laboratory and pathogenic *Escherichia coli* strains. *BMC Microbiol*. **9**, 252. https://doi.org/10.1186/1471-2180-9-252 (2009).

6. Jinneman, K. C., Yoshitomi, K. J. & Weagant, S. D. Multiplex real-time PCR method to identify Shiga toxin genes *stx1* and *stx2* and *Escherichia coli* O157:H7/H- serotype. *Appl. Environ. Microbiol*. **69**, 6327-6333 (2003).

7. Steyert, S. R. *et al*. Comparative genomics and *stx* phage characterization of LEE-negative Shiga toxin-producing *Escherichia coli*. *Front. Cell. Infect. Microbiol*. **2**, 133. https://doi.org/10.3389/fcimb.2012.00133 (2012).

8. Thuraisamy, T. & Lodato, P. B. Influence of RNase E deficiency on the production of *stx2*-bearing phages and Shiga toxin in an RNase E-inducible strain of enterohaemorrhagic *Escherichia coli* (EHEC) O157:H7. *J. Med. Microbiol*. **67**, 724-732 (2018).

9. Hall, B.G. *et al*. Growth rates made easy. *Mol. Biol. Evol*. **31**, 232-238 (2014).
